# The protective effect of allicin on myocardial ischemia-reperfusion by inhibition of Ca^2+^ overload-induced cardiomyocyte apoptosis via the PI3K/GRK2/PLC-γ/IP3R signaling pathway

**DOI:** 10.18632/aging.203375

**Published:** 2021-08-03

**Authors:** Tong Gao, Peng Yang, Dongliang Fu, Mengru Liu, Xinyi Deng, Mingjing Shao, Jiangquan Liao, Hong Jiang, Xianlun Li

**Affiliations:** 1 Graduate School of Peking Union Medical College, Chinese Academy of Medical Sciences, Beijing 100730, China; 2 Department of Integrative Medicine Cardiology, China-Japan Friendship Hospital, Beijing 100029, China; 3 Peking University China-Japan Friendship School of Clinical Medicine, Beijing 100029, China

**Keywords:** allicin, apoptosis, PI3K/Akt pathway, myocardial ischemia/reperfusion, gene expression profiling, calcium signaling pathway, GRK2, GPCR

## Abstract

Purpose: To investigate the protective effect and mechanism of allicin on myocardial ischemia-reperfusion (MI/R) injury.

Methods: We investigated the mechanisms by which allicin attenuated the MI/R injury by focusing on phosphoinositide 3-kinase, G protein coupled receptor kinases 2, phospholipase Cγ and cardiomyocyte apoptosis. Sixty male mice were randomly assigned into three groups: repeated MI/R (model), sham-operated (control), and MI/R+ allicin group (allicin). Ultrasound examination was used to examine the cardiac function. Masson staining was used to evaluate the myocardial infarct area. TUNEL assay was performed to examine the anti-apoptotic effect of allicin. Differentially expressed genes (DEGs) and pathways were analyzed by mRNA microarray analysis. Immunofluorescence staining and western blot were carried out to detect the effect of allicin on the PI3K. A pan-PLC activator, m-3M3FBS, was applied to investigate whether allicin induced cardiomyocyte apoptosis was via the GRK2/PLC/IP3R signaling pathway.

Results: Masson staining and the TUNEL assay revealed that allicin reduced infarct size and played an anti-apoptotic role in M/IR. Ultrasound examination revealed that allicin improved cardiac function after M/IR injury. Gene ontology analysis indicated that the calcium signaling pathway and PI3KCA(PI3K) were selected. Immunofluorescence staining and western blot exposed that PI3K was activated by allicin during MI/R injury. Fura-2AM staining revealed that the PI3K -mediated GRK2/PLC-γ/IP3R pathway may be involved in the protective effect of allicin on MI/R injury.

Conclusions: Allicin has a protective effect on MI/R injury. This effect might be associated with the inhibition of Ca^2+^ overload-induced apoptosis and the inhibition of the PI3K -mediated GRK2/PLC-γ/IP3R signaling pathway.

## INTRODUCTION

Myocardial ischemia-reperfusion (MI/R) injury is a common disease and continues to have high morbidity and mortality in the world [1, 2]. MI/R injury is considered reperfusion injury in patients who suffered from cardiovascular surgery and may develop into some severe cardiovascular diseases, such as hypertension, atherosclerosis, and heart failure [3–5]. Emerging evidence indicates that MI/R injury is a complicated pathophysiological process associated with an intense inflammatory reaction, impaired metabolism, and active cardiomyocyte apoptosis [6–8]. Remarkably, it has been shown that MI/R occurs when an interruption temporarily blocks blood flow to a part of the heart and results in cardiomyocyte apoptosis. Apoptosis, or active cardiomyocyte death, is recognized as the primary cause of MI/R [9]. Intracellular Ca^2+^ overload is considered to be a significant cause of ischemic myocardial cell injury and apoptosis [10, 11]. Additionally, various studies have confirmed that intracellular calcium overloading could lead to mitochondrial dysfunction, which is crucial for facilitating cardiomyocyte apoptosis in MI/R injury [12]. Therefore, it is urgent to explore some anti-apoptotic pretreatments for ischemia diseases.

Allicin (allyl 2-propenethiosulfinate or diallyl thiosulfinate) is a natural compound containing sulfur obtained from garlic, a drug and food in China [13]. The clinical application of allicin gained considerable attention in recent decades. Accumulated biochemical data have shown that allicin has numerous helpful effects, including improving immunity, antioxidant, anti-inflammatory, antibacterial [14, 15].

Besides, emerging evidence indicates that conformational transformation of GPCR (AT1R) leads to the subsequent dissociation of heterotrimer G protein on stimulation [16, 17]. GRK2 is a multifunctional protein kinase of the GRK family that can phosphorylate AngII-occupied GPCR (AT1R) [18–20]. The structure of GRK2 consists of three modularized domains: RH domain, a central catalytic domain, and PH domain in which GRK2 interacts with the G protein. Given that GPCR is a heterotrimer that releases Gβγ and Gα on activation [21–23]. GRK2 could be phosphorylated by various non-GPCR substrates and kinases at different residues [24, 25]. Indeed, the rapid phosphorylation of GRK2 on its residues could accelerate its degradation and impairs its interaction with G protein (Gβγ and Gα) [14, 23, 26]. A recent study has highlighted that PI3K activation could promote GRK2 S670 phosphorylation. The phosphorylation of GRK2 on S670 residue significantly impairs the interaction of GRK2 with Gβγ subunits [27, 28]. It has been previously shown that the release of Gβγ ultimately leads to the activation of phospholipase Cγ(PLCγ) [29, 30]. The activation of PLCγ produces a biophysical composition of three different inositol 1, 4, 5-trisphosphate receptors (IP3Rs), which trigger continuous Ca^2+^ release from the endoplasmic reticulum (ER) to mitochondrial through mitochondrion-associated ER membrane (MAM) [31, 32]. As reported previously, allicin plays an essential role in activating phosphoinositide 3-kinase (PI3K) [33]. The present study was conducted to examine the protective effect of allicin on MI/R injury and investigate whether the mechanism is correlated with PI3K/GRK2/PLCγ induced suppression of Ca^2+^ overload and mitochondrial dysfunction.

## MATERIALS AND METHODS

### Materials

Allicin formulation (5 mg/ml) was purchased from Shanghai Yuanye Biological Ltd. (Shanghai, CN) and given 5 mg/ml. Antibodies against Bax, cleaved caspase-3/9, and BCL-2 were purchased from Abcam (Cambridge, UK). All other reagents were purchased from Cell Signaling Technology (Danvers, MA, USA). Anti-β-actin antibody was purchased from Sigma-Aldrich (St. Louis, MO, USA). All the reagents for immunohistochemistry (rabbit polyclonal 1:200; rabbit polyclonal antibody; Novus Biologicals, Littleton, CO, USA).

### Animals and experimental grouping

A total of sixty male Albino Wistar rats, weighing 270–330, were obtained from the experimental animal center of Henan Province and fed in a 25°C air-conditioned room under a 12-hour light/dark cycle on standard food and water. Mice were divided into two equal groups (*n* = 30): Myocardial ischemia/reperfusion (IR) group served as MI/R model group, Myocardial ischemia/reperfusion (IR) + allicin (1.88 mg allicin/kg) group served as allicin group. Treatment of allicin was administered orally via gavage of allicin (1.88 mg allicin/kg) 12 hours before the induction of ischemia.

### MI/R injury model

The experimental mice were anesthetized with 1% pentobarbital sodium (50 mg/kg) and fixed in a supine position, and the skin of the neck and chest was disinfected. The skin of the chest was clean, and a left thoracotomy was conducted to uncover the heart. (frequency 60 times/min, tidal volume 20 ml/kg, breathing ratio 1.5:1). Tracheal intubation and mechanical ventilation were performed connected to help the mice to breath. The left thoracotomy was conducted to expose the heart (between the 3rd and 4th ribs). After thoracotomy, the left anterior descending branch of the coronary artery (LAD) was rapidly ligated using a silk thread for 30 minutes of ischemia treatment. After 30 min ischemia, the heart was perfused for 3 h. Then observe the color alternations in the ischemic area, and reperfusion was achieved via releasing the knot. Mice in the sham group were operated the same way as those in the MI/R group but without ligating the LAD branch.

### Fluo-2/AM measurements of intracellular-free Ca^2+^

The cardiomyocytes were seeded into 6-well culture plates (3 × 10^5^ cells/well) and then exposed to Fluo-2/AM (Molecular Probes, USA) for 30–40 min at 37°C in the dark, then transferred into a chamber placed on an inverted Nikon Diaphot microscope (Diaphot; Nikon; Tokyo, Japan). Next, illuminated the formulation with the exciting light of a 488 nm light and 488 nm (model D104, Photon Technology International, Inc., South Brunswick, NJ, USA). A photon-counter was used to identify the fluorescence signal and variations. The fluorescence measurement of Ca^2+^ was determined and verified at 10 Hz (Photon Technology). Variations in the emission fluorescence intensity were taken as changes in Ca^2+^.

### Isolation of cardiomyocytes

Primary cultures of cardiomyocytes were obtained from Albino Wistar rats. Hearts were being hastily removed, crushed, and disassociated with 0.25% trypsin. Target cells were then plated at a field density of 2 × 10^5^ cells/cm^2^ on 60-mm culture dishes with Dulbecco’s modified Eagle’s medium (DMEM) supplemented with 10% FBS (Bio Whittaker, Walkersville, Md., US). Then culture the cells at 37°C, 95% O_2_, 5% CO_2_ for 24 h. The medium was then replaced with fresh oxygenated DMEM and then cultured the medium under normoxic conditions (95% O_2_ and 5% CO_2_) medium for 6 h.

### RNA-sequencing

Extraction of the total RNA was performed using RNAiso™ Plus (TaKaRa, Dalian, China). First, created a KAPA Stranded RNA-Seq technology through Illumina SBS sequencing Library was created using 1 μg of total RNA per sample. Then, the RNA was cross-checked using Agilent 2100 Bioanalyzer (Agilent Technologies, USA), the quality was assessed by RT-PCR. Finally, the Illumina HiSeq TM2000 (Kangchen Biotech, Shanghai, China) was applied for further sequencing.

### mRNA microarray analysis

Total RNA was isolated according to the manufacturer’s protocol. After examining the extracted RNA’s quality, cDNA was prepared from isolated total RNA samples (150 ng) (Ambion^®^ WT Expression Kit). Hierarchical clustering was carried out based on differentially expressed mRNAs using Cluster_Treeview software from Stanford University ((M. B. Eisen Laboratory, CA, USA). The EdgeR package (Robinson, McCarthy and Smyth, 2010) was used to transform the raw microarray data into expressions. Differentially expressed genes were identified and analyzed by Limma R packets (Teufel et al., 2016). Gene Set Enrichment Analysis (GSEA) was performed separately in each category package (version 2.10.1). The data of the four groups were analyzed statistically. Gene set enrichment of functional GO and KEGG pathways was analyzed using gene screening software and DAVID online tools, revealing critical pathways of differentially expressed genes. Fisher’s exact test was applied to select the significant pathways, and the threshold of significance was considered *p* < 0.05.

### TUNEL staining

The cardiomyocytes were seeded into 6-well culture plates (3 × 10^5^ cells/well) for 24 h. Exposure of cardiomyocytes to H_2_O_2_ for 2 h, cardiomyocytes were fixed for 20 min in 4% paraformaldehyde at 4°C. Then incubated cultured cardiomyocytes with 0.3% fresh hydrogen peroxide in methanol for 30 min at room temperature. After permeabilizing with 0.1% Triton X-100 in 0.1% sodium citrate and 0.2% Triton X-100 solution for 10 min at 4°C, the cells then were incubated in TUNEL reaction mixture (Roche Diagnostics, Laval, QC, Canada) for 1 h at 37°C, then analyzed using a Nikon fluorescence microscope Eclipse (Nikon Eclipse Ti-S; Nikon Ltd.). For each sample, 200 cells were collected and counted to evaluate apoptosis.

### Immunofluorescence staining

The cardiomyocytes were seeded into 6-well culture plates (3 × 10^5^ cells/well) and rinsed with phosphate-buffered saline, then fixed in 4% paraformaldehyde at 4°C overnight. The fixed cardiomyocytes were incubated with 0.3% Triton X-100 in PBS for 20 min. The cardiomyocytes were blocked with 10% normal goat serum in 0.3% Triton X-100 for 1 h and then incubated with primary antibodies overnight at 4°C. After three times washes with PBS, the cells were incubated with Immunofluorescence-labeled secondary antibodies (Alex Flour (R) 488), the cell nuclei were visualized with DAPI (4Ј, 6-diamidino-2-phenylindole dihydrochloride; Molecular Probes, USA). Images were mounted with (Olympus IX70, Tokyo, Japan).

### Measurement of infarct size and Masson staining

At the end of 48 h reperfusion, the hearts were quickly detached and cleaned, and fixed in 4% paraformaldehyde for a minimum of 48 h. Then sliced the heart and stained with hematoxylin, water-soluble aniline blue, and sulfuric acid Magenta. After that, slides were rinsed in running water for 5 minutes and dipped into absolute xylene for 1 min. In the end, they were fixed with a coverslip using DPX mounting. These slices were examined using a microscope with ×500 magnification and HistoQuest image analysis software (TissueGnostics).

### Real-Time qPCR

Total RNA was extracted using Trizol reagent, and the quantitative polymerase chain reaction (qPCR) package compels a real-time PCR machine (Bio-Rad, USA). The standard curve was attained cycle threshold (Ct) by qPCR reaction cycle curve. Fold change = 2^−ΔΔCt^, and data were analyzed with StepOne software (Applied Biosystems, CA, USA). The β-actin gene was evaluated to assess the reference of the RT-PCR.

### Western blot

At the end of 48 h reperfusion, total protein was extracted from the MI area and determined using the BCA protein assay kit (Beyotime). Equal protein samples were separated in 10% SDS-PAGE and transferred onto a polyvinylidene difluoride membrane. After the membranes were incubated with primary antibodies overnight at 4°C, the membranes were incubated with corresponding secondary antibodies for 2 h at RT. The immunoblots were developed using horseradish peroxidase (HRP)-coupled anti-mouse and then identified with enhanced chemiluminescence (Pierce Biotechnology, Rockford, IL, USA). The intensities of the band were analyzed using a Gene Genius Bio Imaging System.

### Statistics

Data were expressed as the mean ± SD. SPSS 20.0 software was applied to analyze the data. Statistical analysis was conducted using one-way ANOVA analysis followed by Bonferroni’s test. *P* < 0.05 was considered statistically significant.

### Ethics approval

The animal use protocol for this study has been reviewed and approved by the Animal Care Welfare Committee, China-Japan Friendship Hospital.

### Availability of data and materials

The datasets used and analyzed during the current study are available from the corresponding author on reasonable request.

## RESULTS

### Allicin improved cardiac function in mice after myocardial IR injury

**Figure 1:**
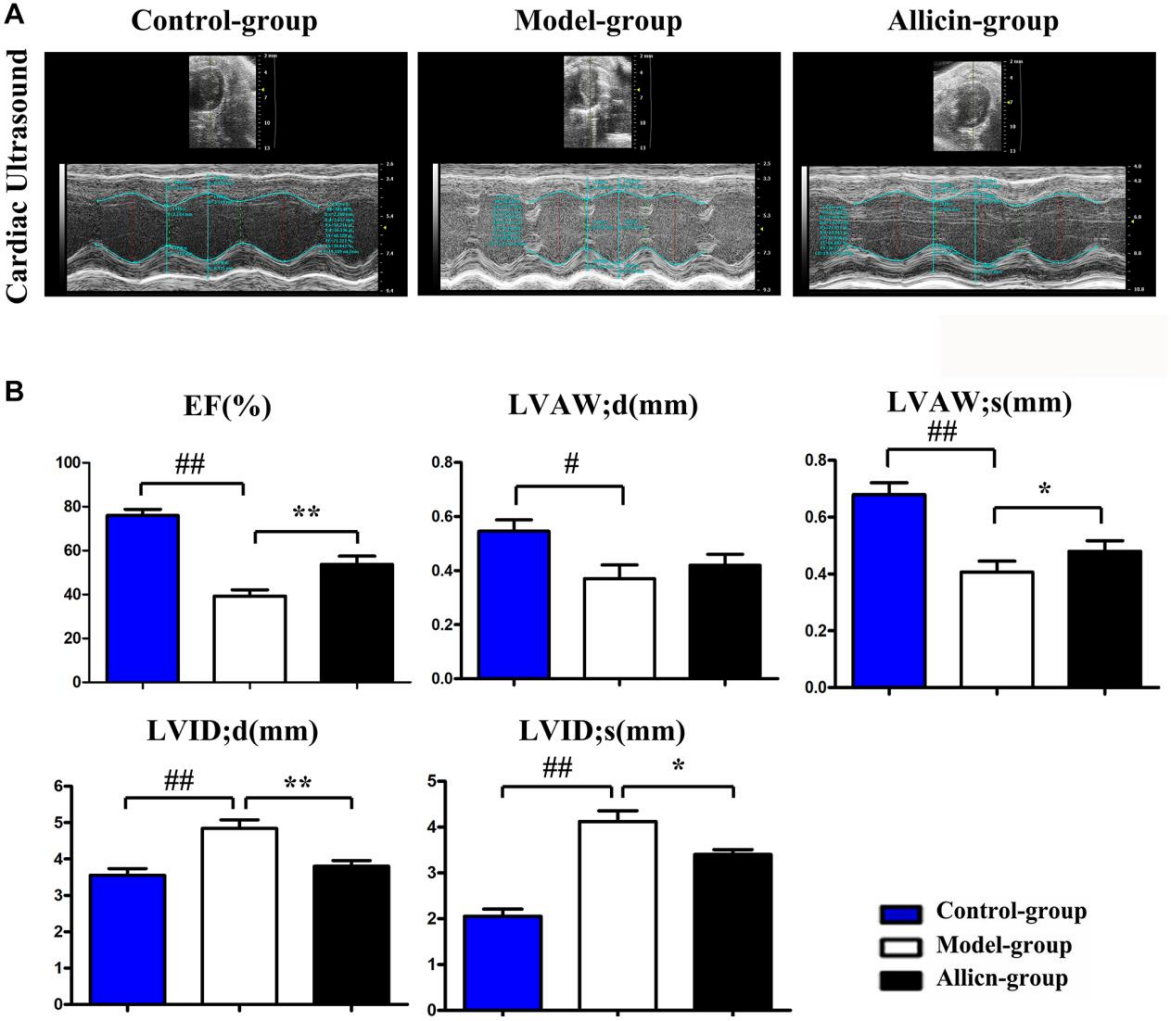
**Allicin improved cardiac function in mice after myocardial IR injury.** (**A**) Ultrasound imaging of mice in the control group, model group, and allicin group. (**B**) The LVEF, LVAW; d, LVAW; s LVDI; d and LVID; s of mice in each group. Data are presented as mean ± SD. ^**^P < 0.05 model vs. allicin group; ^##^*P* < 0.05 model vs. control group. LVAW;d, left ventricular anterolateral wall diameter. LVEF, left ventricular ejection fraction; LVID;d, left ventricular internal diastolic diameter.

To examine the effects of allicin on myocardial IR injury in mice, we performed an ultrasound examination to evaluate the left ventricular wall thickness and cardiac function at day 24. Figure 1A demonstrated the two-dimensional B-mode high-resolution ultrasonographic imaging from different groups. Compared with the control group, LVEF, LVAW; d, and LVAW; s in the model group were lower, while the LVDI; d and LVID; s values were higher (all *P* < 0.05). In the allicin treatment group, allicin could elevate the decrease in LVEF, LVAW; d, and LVAW; s (Figure 1B); however, allicin merely exhibited statistically significant on LVEF and LVAW; s (allicin vs. model). Furthermore, allicin diminished the increase in LVDI; d and LVID; s compared with the model group (Figure 1B, *P* < 0.05). These results implied that allicin could improve cardiac function in mice after myocardial IR injury.

### Effect of allicin on myocardial infarct size and TUNEL-positive cells

**Figure 2:**
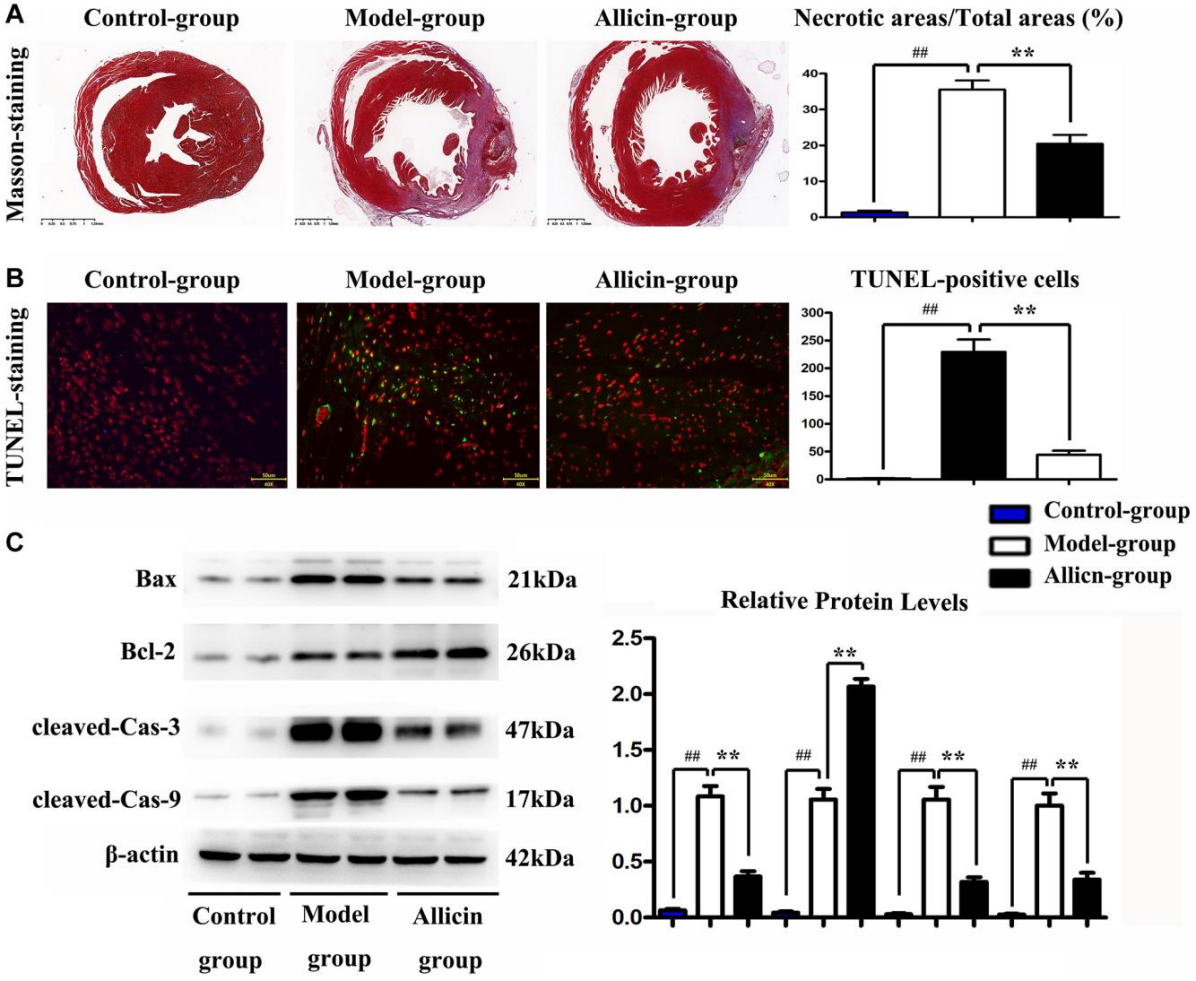
**Allicin treatment reduced infarct size after M/IR and played the anti-apoptotic role in M/IR injury.** (**A**) Masson staining to dye the heart of mice for evaluating the myocardial infarct area after the reperfusion, and the myocardial infarct size in the allicin group was fewer than that in the MI/R group. (**B**) The TUNEL staining, and the quantitative analysis revealed that very few TUNEL-positive cells were detected in the allicin group. (*P* < 0.05, Model vs. Allicin) (**C**) allicin treatment reduced Bax and cleaved caspase-3/9 protein expression and enhanced BCL-2 protein expression by western blot and the quantitative analysis. (*P* < 0.05, Model vs. Allicin).

To assess the therapeutic implications of allicin in MI/R injury, we performed Masson staining to dye the heart of mice for evaluating the myocardial infarct area after the reperfusion. As shown in Figure 2A, we found that the myocardial infarct size turned out to be more significant in the MI/R group (compared with the control group, *P* < 0.05), while the allicin treatment significantly diminished this increase (compared with the MI/R group, *P* < 0.05). Furthermore, we carried out the TUNEL assay to examine the anti-apoptotic effect of allicin. The inhibition of apoptosis by allicin was assessed by the TUNEL staining and was demonstrated in Figure 2B; very few TUNEL-positive cells were detected in the allicin group. The quantitative analysis of TUNEL-positive cells was consistent with the TUNEL assay (*P* < 0.05). Besides, allicin treatment also reduced Bax and cleaved caspase-3/9 protein expression and enhanced BCL-2 protein expression by western blot. (Figure 2C). The quantitative analysis of relative protein levels showed a similar tendency as the western blot. (*P* < 0.05) To the end, these results showed that allicin treatment reduced infarct size after M/IR and played an anti-apoptotic role in M/IR injury.

### Microarray analysis exposed the regulation of the calcium ion pathway, and gene PI3KCA (PI3K) might affect the allicin-induced MI/R protection

**Figure 3:**
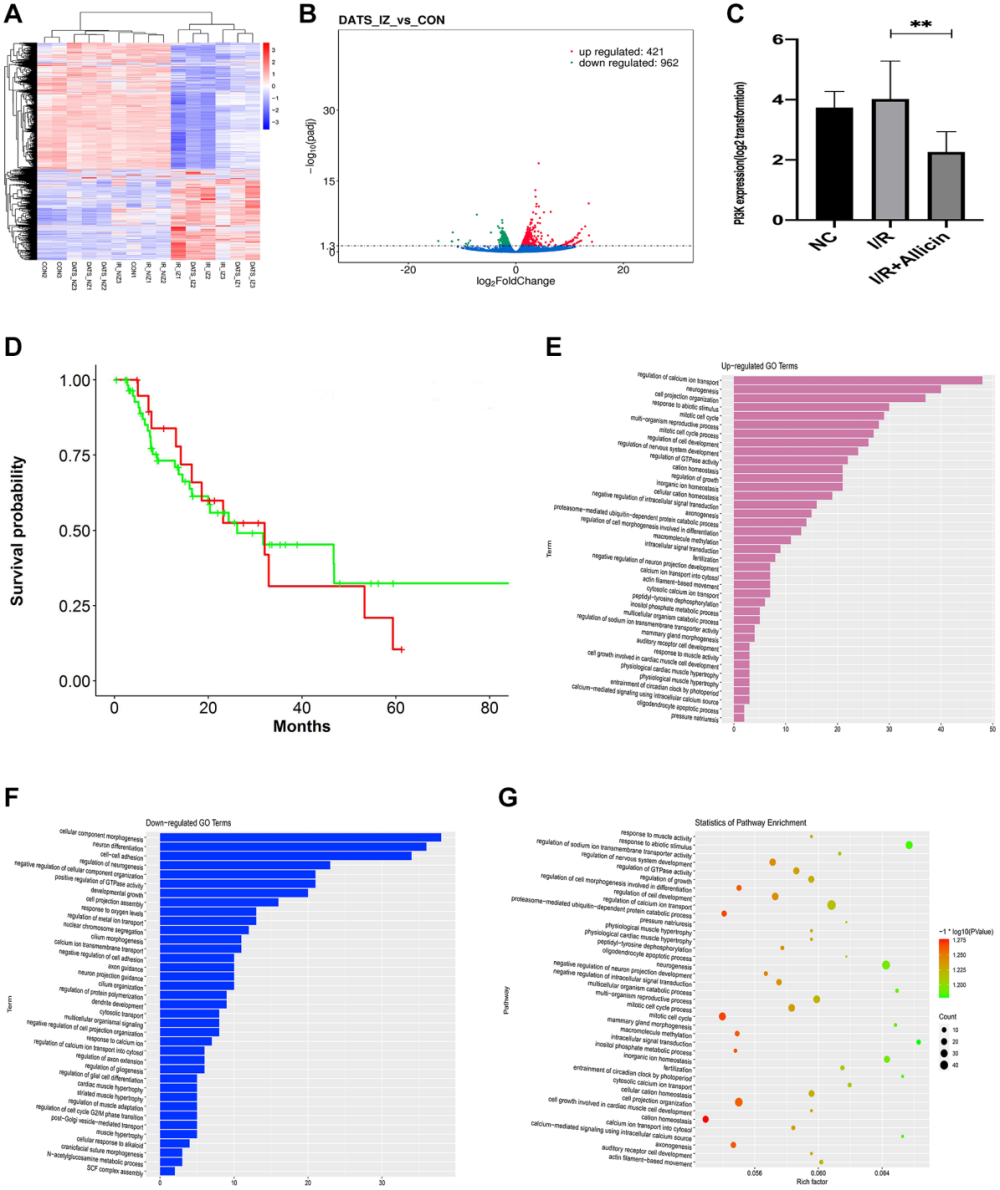
**Microarray analysis exposed the regulation of the calcium ion pathway, and gene PI3KCA (PI3K) might influence the allicin-induced MI/R protection.** (**A**) The hierarchical clustering analysis exhibited an intense distinction on differentially expressed mRNAs in MI/R+ Allicin as exhibited in the heat map. (**B**) The volcano plot exhibited an intense distinction on differentially expressed mRNAs in MI/R+ Allicin as exhibited in the heat map. (**C**) The qPCR analysis revealed that the level of PI3KCA(PI3K) was significantly decreased in MI/R+ Allicin tissues compared with I/R tissues. (*P* < 0.05, MI/R vs. Allicin). (**D**) The receiver operating characteristic (ROC) curve revealed that the expression level of PI3KCA is associated with a higher overall survival rate. (**E**) The annotations of Gene Ontology (GO) found 39 upregulated pathways. (**F**) The annotations of Gene Ontology (GO) found 37 downregulated pathways (Figure 3E). (**G**) The partial results of the KEGG pathways.

To clarify the mechanism of allicin-induced MI/R protection, we extracted RNA from NC, MI/R, and MI/R+ Allicin group to perform mRNA microarray analysis. As shown in Figure 3A, 203 up-regulated differentially expressed genes (DEGs) and 218 down-regulated DGEs were obtained using criteria of |logFC|>2, Padj<0.05 among the three groups, hierarchical clustering analysis exhibited an intense distinction on differentially expressed mRNAs in MI/R+ Allicin as indicated in the heat map. As shown in Figure 3B, a volcano plot was created to screen out the distribution of DEGs between MI/R+ Allicin and MI/R group. A gene named phosphatidylinositol-4; 5-bisphosphate 3-Kinase catalytic subunit alpha (PI3K) was down-regulated in the MI/R+ Allicin group was selected in our study. To further evaluate whether PI3KCA(PI3K) was downregulated in MI/R+ Allicin, we carried out qPCR to analyze the expression of PI3KCA(PI3K) in MI/R+ Allicin, NC, and MI/R tissues. The PI3KCA(PI3K) level was significantly decreased in MI/R+ Allicin tissues compared with I/R tissues. (Figure 3C, *P* < 0.05). Besides, we carried out the receiver operating characteristic (ROC) curve to evaluate the diagnostic value of the PI3KCA level. We found that the expression level of PI3KCA is associated with a higher overall survival rate, indicating PI3KCA might be an excellent diagnostic marker for MI/R injury (Figure 3D). As shown in Gene Ontology (GO) annotations in Figure 3E–3F, we found 39 upregulated pathways (Figure 3E), including regulation of calcium ion transport, calcium ion transport into the cytosol, cytosolic calcium ion transport, and peptidyl-tyrosine dephosphorylation. Furthermore, we also found 37 downregulated pathways (Figure 3F), including cellular component morphogenesis, neuron differentiation, and cell-cell adhesion. Similarly, partial results of the KEGG pathways were exhibited in Figure 3G. Considering the function and the frequency of the gene in the enriched pathway associated with MI/R injury, we finally chose PI3KCA (PI3K) and calcium ion transport signaling pathways for further investigation.

### Allicin increased p-PI3K protein expression

**Figure 4:**
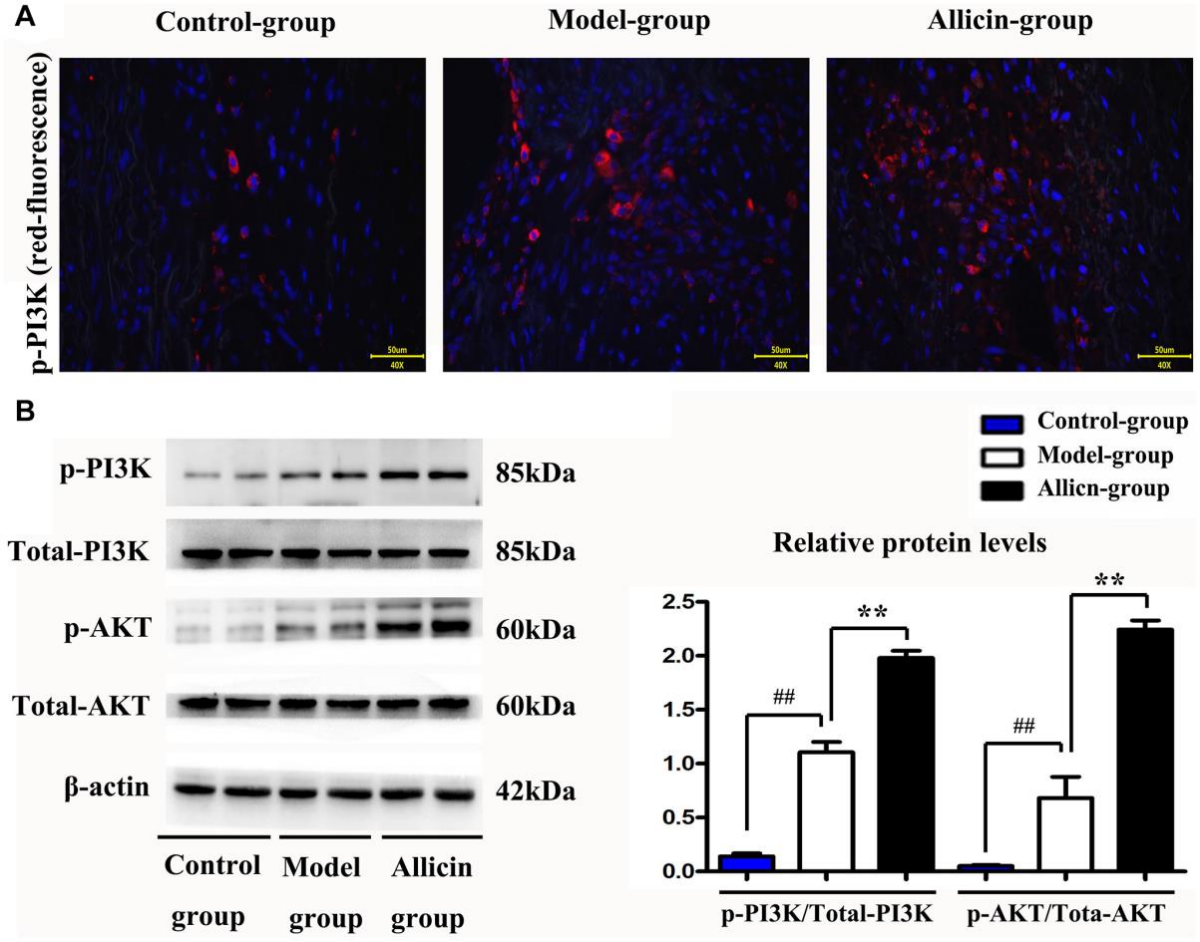
**Allicin increased p-PI3K protein expression.** (**A**) Immunofluorescence staining revealed that allicin could increase the level of p-PI3K in cardiomyocytes. (**B**) The quantitative analysis and western blot analysis revealed that allicin treatment caused a marked increase in the phosphorylation of Akt and PI3K compared with the MI/R group. (*P* < 0.05, Model vs. Allicin).

As shown in Figure 4A, the MI/R group showed an increased p-PI3K level (compared with the control group, *P* < 0.05), while allicin significantly increased the level of p-PI3K (compared with the MI/R group, *P* < 0.05) in myocardial tissues. To further confirm the effect of allicin treatment on the PI3K/Akt pathway, we assessed related proteins of p-Akt and p-PI3K by western blot analysis. Figure 4B showed that the protein expression of p-Akt and p-PI3K was enhanced by MI/R, and allicin treatment showed a more significant effect than the model. Our quantitative analysis further confirmed that allicin treatment caused a marked increase in the phosphorylation of Akt and PI3K compared with the MI/R group (Figure 4B, *P* < 0.05). Consistent with microarray analysis and bioinformatics analysis, PI3K was activated by allicin pretreatment during MI/R injury in the present study.

### Allicin repressed the expression of p-GRK2, p-CaMKII, p-PLC-γ, and p-IP3R during MI/R injury

**Figure 5:**
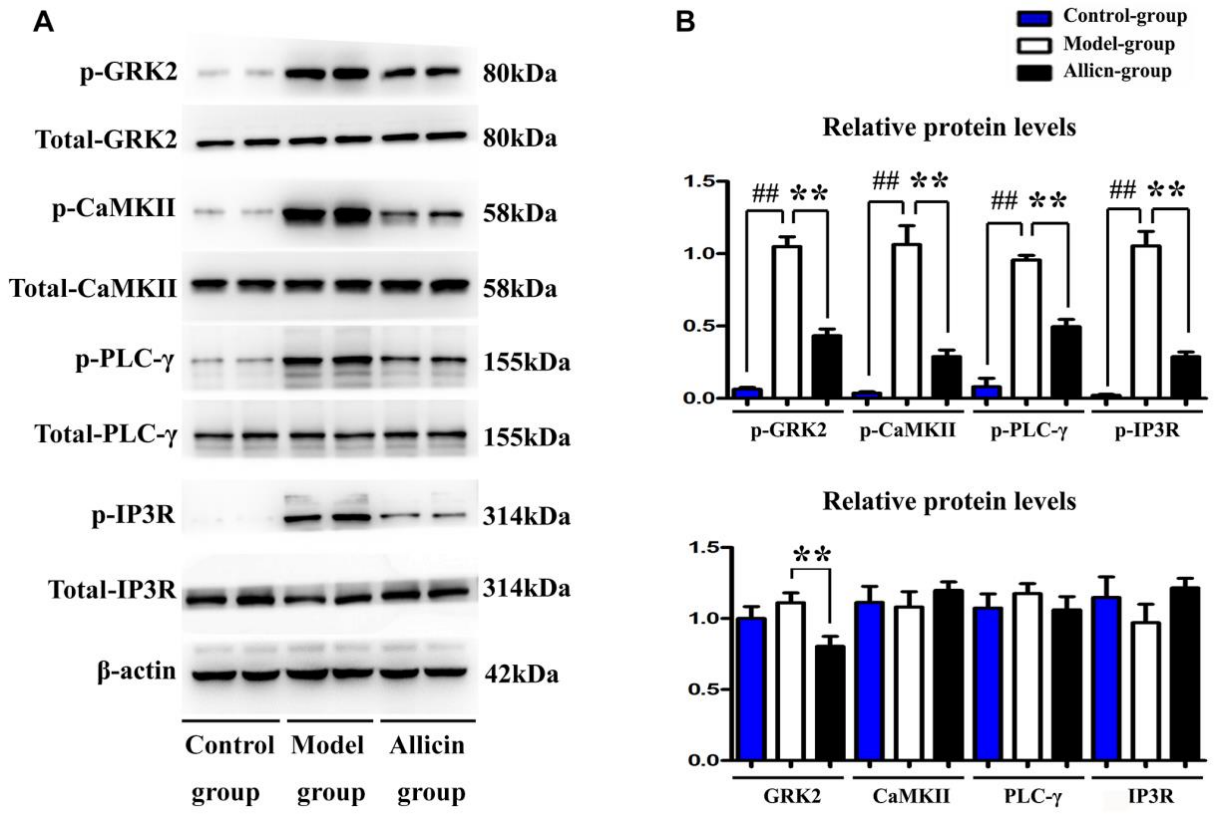
**Allicin repressed the expression of p-GRK2, p-CaMKII, p-PLC-γ, and p-IP3R during MI/R injury.** (**A**) The western blot analysis revealed that MI/R injury could enhance the expression of p-GRK2, p-CaMKII, p-PLC-γ, and p-IP3R, whereas the pretreatment with allicin can reduce the expression of p-GRK2, p-CaMKII, p-PLC-γ, and p-IP3R. (**B**) The quantitative analysis revealed that MI/R injury could enhance the expression of p-GRK2, p-CaMKII, p-PLC-γ, and p-IP3R, whereas the pretreatment with allicin can reduce the expression of p-GRK2, p-CaMKII, p-PLC-γ, and p-IP3R. (*P* < 0.05, Model vs. Allicin).

To explore the mechanism associated with allicin’s inhibitory effect on Ca^2+^ overload and mitochondrial dysfunction, we evaluated p-GRK2, p-CaMKII expression p-PLC-γ, and p-IP3R, which has been confirmed to regulate Ca^2+^ release and mitochondrial dysfunction. As shown in Figure 5A, MI/R injury can enhance p-GRK2, p-CaMKII, p-PLC-γ, and p-IP3R (compared with the control group, *P* < 0.05), whereas the pretreatment with allicin can block the increase of p-GRK2, p-CaMKII, p-PLC-γ, and p-IP3R. Our quantitative analysis of relative protein levels further confirmed that the expression levels of p-GRK2, p-CaMKII, p-PLC-γ, and p-IP3R were decreased in the Allicin group as compared with the MI/R group (Figure 5B, *P* < 0.05). Additionally, total GRK2 was decreased by allicin (compared with model group, *P* < 0.05), which agreed with the notion that allicin-induced PI3K activation might contribute to the degradation of GRK2 [34]. Moreover, the phosphorylation of GRK2 on S670 residue significantly impairs the interaction of GRK2 with Gβγ subunits. In brief, our findings were consistent with the findings from previous studies that PI3K activation could promote GRK2 S670 phosphorylation, which might impair the interaction of GRK2 with Gβγ subunits [35, 36]. And the release of Gβγ ultimately leads to the activation of phospholipase Cγ(PLCγ) that could produce a biophysical composition of three different inositol 1, 4, 5-trisphosphate receptors (IP3Rs), which triggers continuous Ca^2+^ release [37]. In brief, these results indicated that allicin pretreatment could repress the activation of the PI3K induced GRK2/PLC-γ/IP3R signal pathway during MI/R injury.

### Effect of allicin on Ca^2+^ increase in MI/R injury via PI3K -mediated GRK2/PLC-γ/IP3R pathway

**Figure 6:**
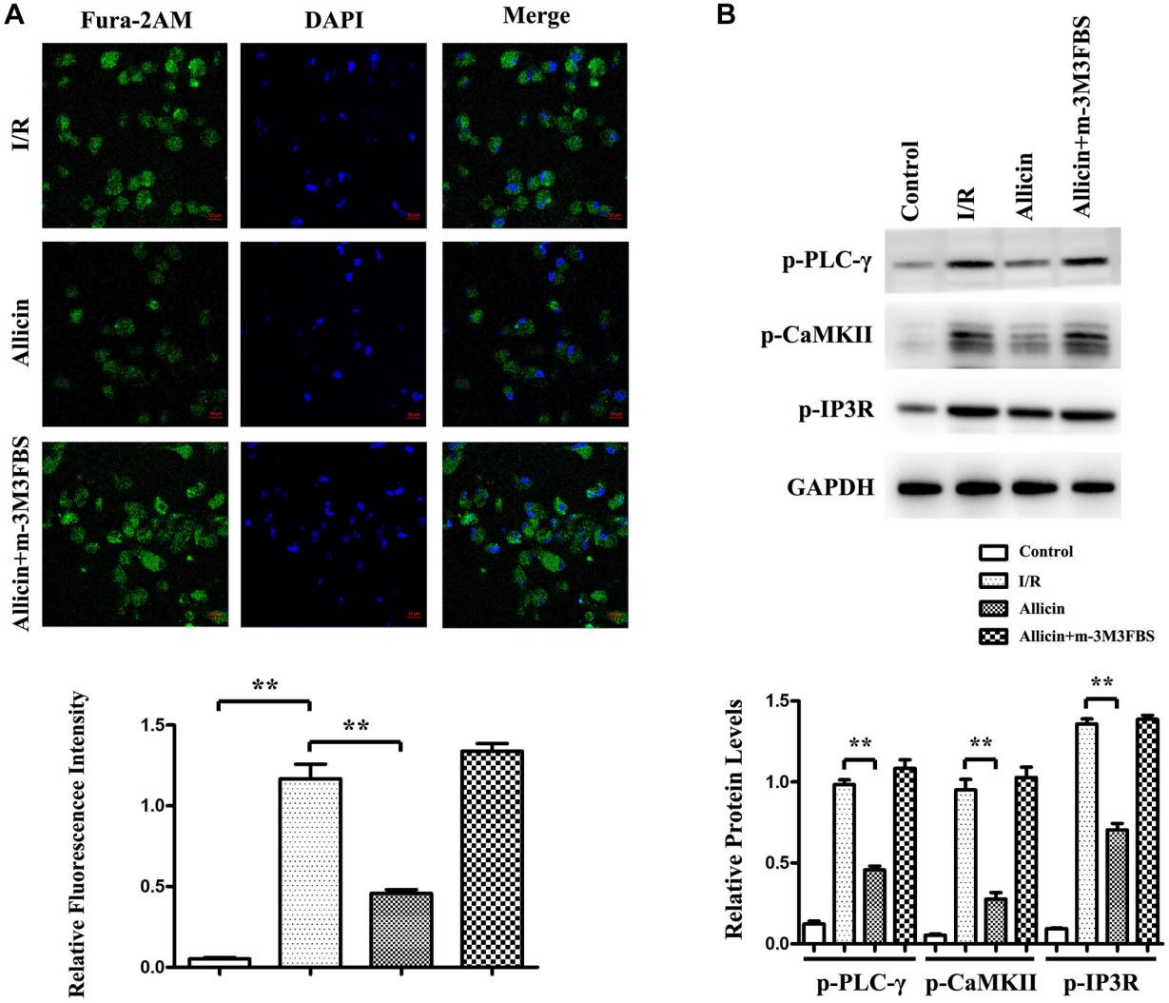
**Effect of allicin on Ca^2+^ increase in MI/R injury via PI3K -mediated GRK2/PLC-γ/IP3R pathway.** (**A**) The cardiomyocytes from MI/R, MI/R+ Allicin, and MI/R+ Allicin+ m-3M3FBS were stained with Fura-2AM dye, showing allicin decreased intracellular Ca^2+^ release during MI/R injury, and the pan-PLC activator m-3M3FBS reversed the decreased intracellular Ca^2+^ release during MI/R. (**B**) The western blot and quantitative analysis revealed that p-CaMKII, p-PLC-γ, and p-IP3R were increased in the MI/R group and reduced in the Allicin group m-3M3FBS could reverse the decrease of p-CaMKII, p-PLC-γ, and p-IP3R expression levels. (*P* < 0.05, Model vs. Allicin).

As we demonstrated that allicin could repress cardiomyocyte apoptosis and activate PI3K, which caused the inhibition of GRK2, PLC-γ, and IP3R induced Ca^2+^ release, we then examined the potential mechanism involved. A pan-PLC activator, 2, 4, 6-trimethyl-N-(m-3-trifluoromethylphenyl) benzenesulfonamide (m-3M3FBS), was injected into the culture medium of cardiomyocytes from the MI/R+ Allicin mice group. As shown in Figure 6A, the cardiomyocytes from MI/R, MI/R+ Allicin, and MI/R+ Allicin+ m-3M3FBS were stained with Fura-2AM dye, showing allicin decreased intracellular Ca^2+^ release during MI/R injury, and the pan-PLC activator m-3M3FBS reversed the decreased intracellular Ca^2+^ dismissal during MI/R. Our quantitative analysis of relative fluorescence intensity further confirmed that the Ca^2+^ levels were increased in the MI/R group compared with the control group and decreased in the Allicin group compared with the MI/R group. (*P* < 0.05) In western blot, we found that p-CaMKII, p-PLC-γ, and p-IP3R were increased in the MI/R group and reduced in the Allicin group, m-3M3FBS reversed the decrease of p-CaMKII, p-PLC-γ, and p-IP3R expression levels. The quantitative analysis of relative protein levels showed a similar tendency as the western blot. (Figure 6B, *P* < 0.05). These results suggested that the protective effect of allicin on MI/R injury by PI3K -mediated was via GRK2/PLC-γ/IP3R pathway.

## DISCUSSION

MI/R injury is associated with an amplified myocardial ischemic area at risk and infarct size [38, 39]. In this present study, our MI/R injury model showed significantly increased myocardial infarct size. In contrast, the myocardial infarct size in the Allicin group was significantly improved, suggesting that the injured myocardium was enhanced in mice pretreated with allicin and that allicin had a protecting effect on MI/R injury. Furthermore, we performed ultrasound examination to evaluated whether allicin improved the cardiac function in mice after myocardial IR injury. The results revealed that allicin could increase LVEF, LVAW; d, and LVAW; s and decreased LVDI; d and LVID; s, implying that allicin could improve cardiac function in mice after myocardial IR injury.

Although several pathogenic pathways converge to accelerate MI/R development, the necessary inducement seems to be the death of active cardiomyocytes in response to MI/R injury [40]. Intracellular Ca^2+^ overload is considered to be a significant cause of ischemic myocardial cell injury and apoptosis [41]. The results of our study showed that very few TUNEL-positive cells were detected in the Allicin group. The expressions of Bax and cleaved caspase 3/9 in the MI/R group were higher than those in the Allicin group. The expressions of Bax and cleaved caspase 3/9 were significantly reduced after allicin pretreatment. Besides, the expression of Bcl-2 was lower than that in the Allicin group and was enhanced after allicin pretreatment. These results implied that allicin could attenuate infarct size and apoptosis after myocardial injury.

Our study carried out microarray analysis and bioinformatics analysis to exhibit the differentially expressed genes and pathways of MI/R injury. The hierarchical clustering analysis between MI/R patients, normal controls, and MI/R + Allicin (Allicin) patients found 203 up-regulated differentially expressed genes (DEGs) and 218 down-regulated DGEs using criteria of |logFC|>2, Padj<0.05. A gene named PI3KCA(PI3K), downregulated in the Allicin group, was selected in our study. Our qRT-PCR analysis revealed that the level of PI3KCA(PI3K) was significantly decreased in Allicin tissues as compared with MI/R tissues (*P* < 0.05). The receiver operating characteristic (ROC) curve exposed that the expression level of PI3KCA is associated with a higher overall survival rate, indicating PI3KCA might be an excellent diagnostic marker for MI/R injury. Furthermore, our pathway analysis revealed 39 upregulated pathways and 37 downregulated pathways. The gene set enrichment analysis (GSEA) showed that the PI3KCA was functionally enriched in regulating the calcium ion transport signaling pathway. Considering the function and the frequency of the gene in the enriched pathway associated with MI/R injury, we finally chose PI3KCA (PI3K) and calcium ion transport signaling enriched pathway for further investigation.

To gain additional insight into the effects of allicin on PI3KCA (PI3K) signaling during MI/R injury, we analyzed the PI3K signaling pathway *in vitro*. Consistent with microarray analysis and bioinformatics analysis, the immunofluorescence staining, and western blot revealed that the PI3K was activated by allicin during MI/R injury in the present study.

GRK2 is a multifunctional protein kinase of the GRK family that can phosphorylate GPCR (AT1R) [23, 20]. The structure of GRK2 consists of three modularized domains: RH domain, a central catalytic domain, and PH domain in which GRK2 interacts with the G protein [27]. Given that GPCR is a heterotrimer that releases Gβγ and Gα on activation [23, 24]. In addition to triggering GPCR phosphorylation, GRK2 could be phosphorylated by various non-GPCR substrates and kinases at different residues [24]. Indeed, the rapid phosphorylation of GRK2 on its residues could accelerate its degradation and impairs its interaction with G protein (Gβγ and Gα) [23]. A recent study has highlighted that PI3K activation could promote GRK2 S670 phosphorylation [28]. The phosphorylation of GRK2 on S670 residue significantly impairs the interaction of GRK2 with Gβγ subunits [27]. It has been previously shown that the release of Gβγ ultimately leads to the activation of phospholipase Cγ(PLCγ) [29]. The activation of PLCγ produces a biophysical composition of three different inositol 1, 4, 5-trisphosphate receptors (IP3Rs), which trigger continuous Ca^2+^ release from the endoplasmic reticulum (ER) to mitochondrial through mitochondrion-associated ER membrane (MAM) [32, 41]. Our data confirmed that allicin contributed to the activation of phosphoinositide 3-kinase (PI3K). Our results were consistent with the findings from previous studies that allicin could activate PI3K/GRK2/PLC-γ/IP3R signaling pathway. To investigate the mechanism associated with allicin’s suppression of Ca^2+^ overload and mitochondrial dysfunction, we evaluated the GRK2/PLC-γ/IP3R signal pathway *in vivo*. Our results exposed that MI/R injury can enhance the expression of p-GRK2, p-CaMKII, p-PLC-γ, and p-IP3R. In contrast, the pretreatment with allicin can reduce the expression of t-GRK2, p-GRK2, p-CaMKII, p-PLC-γ, and p-IP3R, which were consistent with the findings from previous studies that allicin could activate PI3K mediated GRK2/PLC-γ/IP3R signaling pathway, which had been confirmed to regulate Ca^2+^ release and mitochondrial dysfunction during MI/R injury.

As we demonstrated that allicin repressed cardiomyocyte apoptosis and increased Ca^2+^ induced by GRK2/PLC/IP3R signaling pathway, we then examined the potential mechanism involved. A pan-PLC activator, m-3M3FBS, was injected into the culture medium of cardiomyocytes from the MI/R+ Allicin mice group. The cardiomyocytes from MI/R, MI/R+ Allicin, and MI/R+ Allicin+ m-3M3FBS were stained with Fura-2AM dye, showing allicin decreased intracellular Ca^2+^ increases after MI/R injury and the pan-PLC activator m-3M3FBS induced intracellular Ca^2+^ increases in MI/R. In western blot, we found that p-CaMKII, p-PLC-γ, and p-IP3R were increased in the MI/R group and reduced in the Allicin group m-3M3FBS could reverse the decrease of p-CaMKII, p-PLC-γ, and p-IP3R expression levels. These results suggested that the protective effect of allicin on MI/R injury by PI3K-mediated was via GRK2/PLC-γ/IP3R pathway.

**Figure 7:**
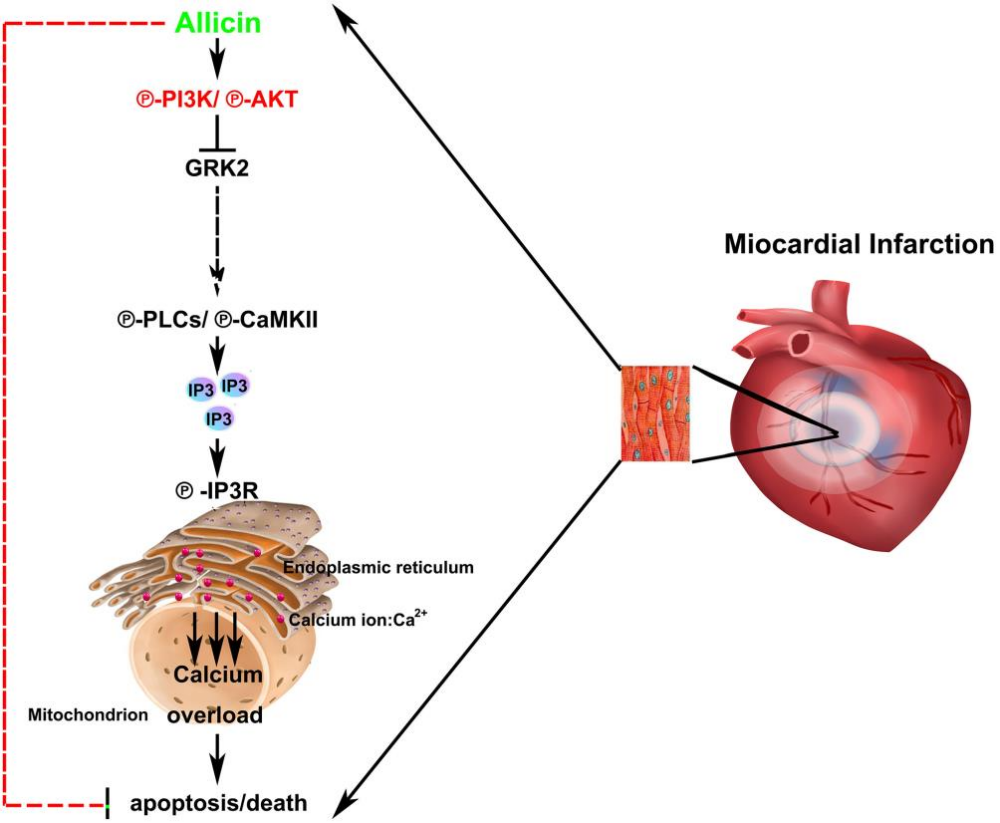
Schematic illustration of the proposed signaling mechanism/pathway underlying allicin’s protective effects.

In summary, our present study extended these studies by a series of *in vivo* and *in vitro* experiments. As shown in Figure 7, it demonstrated for the first time that the protective effect of allicin against MI/R injury was related to the activation of PI3K, GRK2, PLC-γ, and Ca^2+^ overload and a consequent reduction in cardiomyocyte apoptosis. These findings might be significantly more to understand the molecular mechanisms of allicin on heart protection.
